# A Prototype Recombinant-Protein Based *Chlamydia pecorum* Vaccine Results in Reduced Chlamydial Burden and Less Clinical Disease in Free-Ranging Koalas (*Phascolarctos cinereus*)

**DOI:** 10.1371/journal.pone.0146934

**Published:** 2016-01-12

**Authors:** Courtney Waugh, Shahneaz Ali Khan, Scott Carver, Jonathan Hanger, Joanne Loader, Adam Polkinghorne, Kenneth Beagley, Peter Timms

**Affiliations:** 1 Faculty of Science, Health, Education and Engineering, University of the Sunshine Coast, 90 Sippy Downs Dr, Sippy Downs, 4558, Queensland, Australia; 2 Institute of Health and Biomedical Innovation, Queensland University of Technology, 60 Musk Ave, Kelvin Grove, 4059, Queensland, Australia; 3 School of Biological Sciences, University of Tasmania, Private Bag 55, Hobart, Tasmania, 7001, Australia; 4 Endeavour Veterinary Ecology, 1695 Pumicestone Rd, Toorbul, 4510, Queensland, Australia; Midwestern University, UNITED STATES

## Abstract

Diseases associated with *Chlamydia pecorum* infection are a major cause of decline in koala populations in Australia. While koalas in care can generally be treated, a vaccine is considered the only option to effectively reduce the threat of infection and disease at the population level. In the current study, we vaccinated 30 free-ranging koalas with a prototype *Chlamydia pecorum* vaccine consisting of a recombinant chlamydial MOMP adjuvanted with an immune stimulating complex. An additional cohort of 30 animals did not receive any vaccine and acted as comparison controls. Animals accepted into this study were either uninfected (*Chlamydia* PCR negative) at time of initial vaccination, or infected (*C*. *pecorum* positive) at either urogenital (UGT) and/or ocular sites (Oc), but with no clinical signs of chlamydial disease. All koalas were vaccinated / sampled and then re-released into their natural habitat before re-capturing and re-sampling at 6 and 12 months. All vaccinated koalas produced a strong immune response to the vaccine, as indicated by high titres of specific plasma antibodies. The incidence of new infections in vaccinated koalas over the 12-month period post-vaccination was slightly less than koalas in the control group, however, this was not statistically significant. Importantly though, the vaccine was able to significantly reduce the infectious load in animals that were *Chlamydia* positive at the time of vaccination. This effect was evident at both the Oc and UGT sites and was stronger at 6 months than at 12 months post-vaccination. Finally, the vaccine was also able to reduce the number of animals that progressed to disease during the 12-month period. While the sample sizes were small (statistically speaking), results were nonetheless striking. This study highlights the potential for successful development of a *Chlamydia* vaccine for koalas in a wild setting.

## Introduction

Infections by the intracellular bacterium *Chlamydia pecorum* contribute to significant morbidity and mortality in the koala (*Phascolarctos cinereus*). Disease progression can include kerato-conjunctivitis, cystitis, reproductive disease/sterility and blindness; the progression of which, in severe cases, can cause death. An antibiotic treatment regime is currently recommended for mild infections [1], however for koalas affected by severe chlamydial disease, antibiotics alone are not sufficient to cure the clinical signs [1].

In recognition that a reduction in disease may have a positive effect in the conservation of koalas [2, 3], our group has been leading the development of a prototype *C*. *pecorum* vaccine [4–9]. Based on studies which have shown efficacy in animal models (reviewed in Farris and Morrison [10]), the primary component of the *C*. *pecorum* vaccine has been the recombinant proteins derived from the chlamydial Major Outer Membrane Protein (rMOMP). rMOMP is highly immunogenic in humans and animals and has been studied in detail as a vaccine candidate. In the initial studies utilizing this vaccine antigen adjuvanted with an immune stimulating complex, we have shown that this prototype chlamydial vaccine (i) induces long-lasting specific humoral and cell-mediated immune responses in vaccinated koalas [9]; (ii) induces an immune response that can recognize genetically distinct *C*. *pecorum* strains, a capability that natural infection does not appear to have [6]; (iii) induces the production of specific antibodies that are effective in neutralizing *C*. *pecorum in vitro* [9]; and (iv) does not have any apparent deleterious effects on the health of *Chlamydia*-free koalas or koalas with current chlamydial infection and/or disease [8, 11].

In the absence of an established infection challenge model for the koala, further understanding of the efficacy of the vaccine for reducing the risk and impact of chlamydial infection at both the individual and population level is limited. In the current study, we assessed the health outcomes of a cohort of 60 koalas, including 30 animals vaccinated with the prototype *Chlamydia* vaccine within one free-ranging population in South-East Queensland (SEQ), Australia. Vaccinated and control cohorts of animals were then released, monitored for a period of 12 months, and recaptured periodically to compare a range of health parameters between the two groups.

## Materials and Methods

### *Chlamydia* MOMP recombinant preparation

Purified *C*. *pecorum* MOMP from three koala *C*. *pecorum* genotypes (A, F and G) were used as previously described by Kollipara et al. [7].

### Animals and Immunizations

Animals included in the study (n = 60) were part of a larger population-wide study by the Queensland Government Department of Transport and Main Roads (as part of the Moreton Bay Rail Link project), conducted between 2012 and 2015 in the Moreton Bay Region, Queensland, Australia. Criteria for inclusion into the study were animals of breeding age (>1 year) of either sex, with no clinical signs of chlamydial disease, as assessed during the initial capture event by qualified wildlife veterinarians. Animals were randomly assigned to either the vaccinated or control (non-vaccinated) group at initial capture. The vaccinated group (n = 30) received a three-dose regime of the vaccine via the sub-cutaneous route, given at one-month intervals, consisting of the three rMOMP proteins as the antigens (50μg each of MOMP-G, MOMP-A, and MOMP-F) and an Immunostimulating complex adjuvant (50μg, ISC, Zoetis Australia [4]). Following a detailed veterinary health assessment, animals were released with a radio collar or anklet for tracking (Sirtrack). Animals were re-captured at 1 month, 2 months, 6 months, and 12 months for the purpose of (i) additional vaccinations for the vaccine cohort animals only (1 month and 2 months) or (ii) detailed health checks and sampling (2, 6 and 12 months). While 30 animals were originally recruited into each group, unfortunately, only 23 vaccinated and 27 control koalas could be resampled at the six month time point due to animal losses associated with misadventure (e.g. predation, trauma, koala movements outside of study area, or disease). At 12 months, again, further losses had occurred and numbers were considerably reduced in each cohort to 15 vaccinated and 14 control koalas.

All procedures were approved by the University of the Sunshine Coast (USC) Animal Ethics Committee (Animal ethics number AN/A/13/80) and by the Queensland Government (Scientific Purposes Permit, WISP11532912). The trial was performed under the Australian Pesticides and Veterinary Medicines Authority Permit PER 7250.

### Health assessments and sampling

Koalas were located by transect searching (for initial capture) or via telemetry devices (for subsequent captures). The choice of capture technique (flagging vs koala trap) was based on the following: if a koala could be safely (for koala and capture team personnel) captured using a flagging technique (dangling a flag on a pole above the head of a koala to gently coax them to the bottom of the tree), then the flagging technique was employed. If the safety of the koala or koala capture team could not be assured for a conventional flagging capture, then the capture used the koala trap, or was aborted. Veterinary assessments and sampling, while under a short period of anesthesia, were conducted on each animal at 0, 2, 6, and 12 month time-points following their initial capture and veterinary examination. Koalas were anaesthetized by intramuscular injection (quadriceps muscle group) of alfaxalone (Alfaxan CD RTU, Jurox) at a dose rate of approximately 3mg/kg. A 22 or 23 gauge needle was used. Minimal or no restraint was required, and the injection was performed while the koala was in the transport cage. After induction of general anesthesia (approximately 5 minutes in most cases) the koala was removed from the cage and placed on the examination table. Anesthesia was maintained by administration of an isoflurane/oxygen mix delivered by face-mask at between 1.5–2.5% vapor pressure of isoflurane. A full veterinary physical and clinical examination was conducted in accordance with standard veterinary procedures Ultrasound examination of the kidneys, ureters, urinary bladder and the reproductive tract allowed for identification of urogenital tract diseases including cystitis and reproductive-tract cysts in female koalas. Urinalysis was utilized to detect possible kidney or urinary tract disorders, such as cystitis, which is also associated with *Chlamydia*. Chlamydial disease scores were assessed according to the disease scoring criteria outlined in detail in Wan et al. [12]. For the purposes of this study, one set of conjunctival/ocular (Oc) and urogenital (UGT) swabs were collected for *Chlamydia* load determination and a blood sample of up to 5mL was collected from the cephalic vein. This was used for preparation of haematology smears and separation of plasma and serum by centrifugation. During anaesthesia and recovery koalas were constantly monitored by an experienced wildlife veterinary nurse. At the completion of all veterinary procedures, volatile anaesthesia delivery was stopped and the koala was monitored closely until it achieved sternal recumbancy. At this point it was transferred to a transport cage until fully recovered from anaesthesia. When the veterinary team was satisfied that the koala had recovered sufficiently to be released back into the wild safely, it was released by field staff at the point of capture.

### *Chlamydia*-specific IgG plasma response

IgG response was analysed via enzyme-linked immunosorbent assays (ELISAs). ELISAs were performed on plasma samples at 0 and 6 months as per Khan et al. [5], and served as a control to demonstrate that vaccinated koalas produced a specific immune response to the vaccine antigens as previously shown [5].

### *Chlamydia* quantification

Swab samples were stored at -20°C until the DNA was extracted as described by Devereaux et al. [13]. The extracted samples were screened for the presence of *C*. *pecorum* using a diagnostic quantitative real-time PCR (RT-PCR) targeting a 204 bp fragment of the chlamydial 16S rRNA gene. Assays were as described in Marsh et al. [14] except for the PCR mixture containing 1× QuantiTect SYBR Green PCR Master Mix (Qiagen) and 10 μM primers [14] made up to a final volume of 15 μl with PCR-grade water, as well as an increased initial denaturation to 15 mins at 94°C. All reactions were carried out on a Rotor-Gene Q 5-plex HRM platform (Qiagen).

### Statistical analysis

Significant differences between 0 and 6 month IgG antibody titres were evaluated with a Wilcoxon signed-rank test. To evaluate how *C*. *pecorum* infection prevalence and loads differed among vaccinated versus control koalas, Chi-square contingency table analyses were used to compare the changes in *C*. *pecorum* load over time (0 vs. 6 and 0 vs. 12 months), with changes categorized into bands as either, decreasing, stable or increasing (ΔqPCR ≤ -100, -99–99, and ≥ 100 copies/μL respectively). These categories were chosen because small variations of up to 100 copies/μL in qPCR can occur across assays. We conducted this analysis on the raw numbers of koalas within each group (which we considered a conservative analysis, given our sample size), and on the percentage of koalas in each group (which we considered a more sensitive approach, given our sample size). We chose these conservative and sensitive approaches because, though our results are striking, the numerical effect of koala mortalities in the field inflated the Type-II statistical error, limiting detection of statistical significance based on raw data alone. Where appropriate we used Markov chain Monte Carlo simulations to overcome Chi-square statistical issues associated with expected values < 5. Analyses were conducted on both Oc and UGT infections (see Results). All analyses were conducted using Rv3.0.2 (www.r-project.org).

## Results

### Vaccine safety data

All vaccinated animals were monitored for up to 24 hours post-vaccination and given a thorough veterinary health check at 2 months and thereafter at their regular 6-monthly capture and sampling events. There were no short or longer-term adverse events reported due to administration of the vaccine in any of the animals.

### Immune response to vaccination

**Fig 1:**
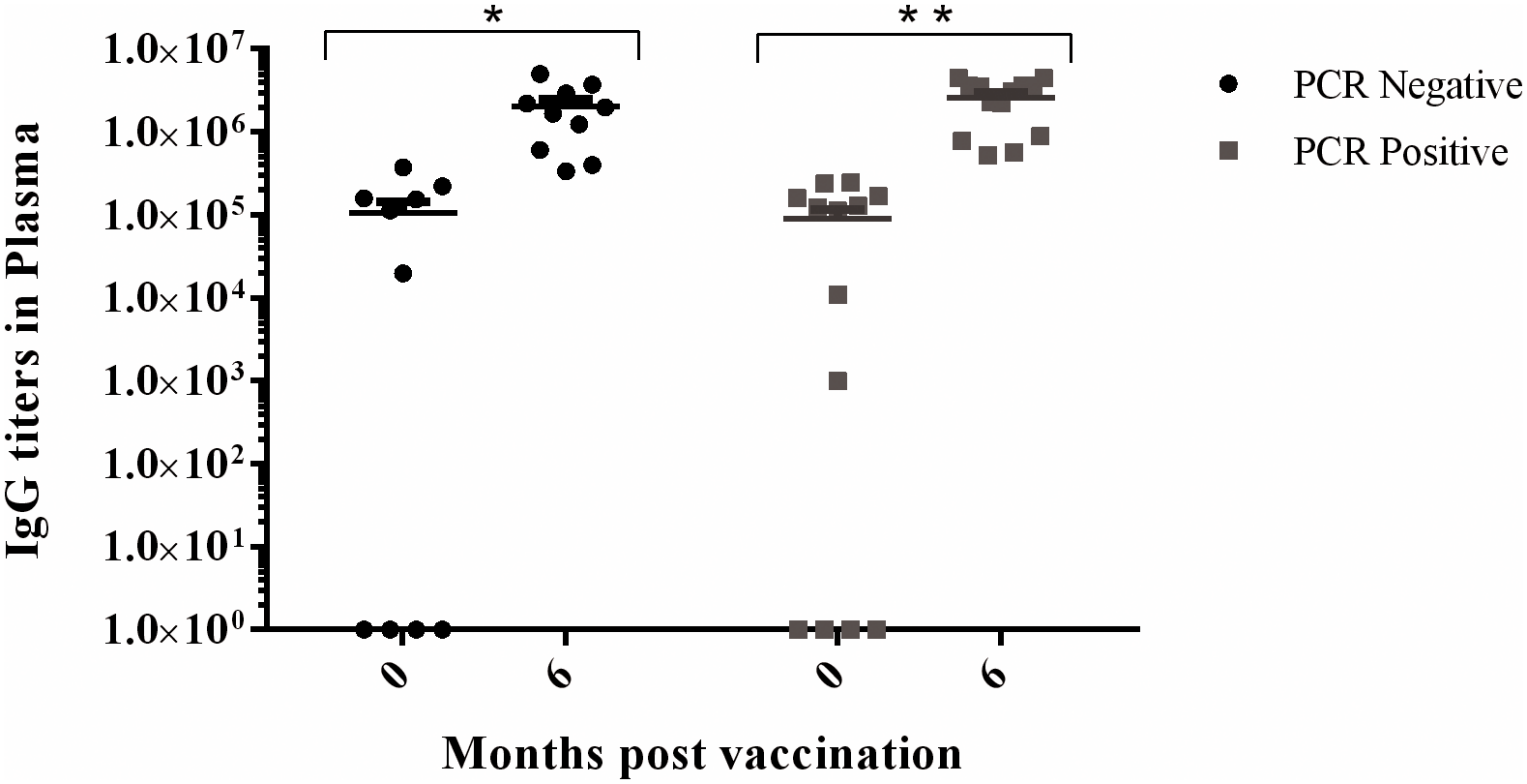
Antibody (IgG) titre response in vaccinated animals at 0 months (pre-vaccination) and 6 months post-vaccination (n = 23): Mean and standard error of IgG titre levels. P < 0.05 *; P < 0.001 **.

We used our *Chlamydia* ELISA to determine plasma IgG antibody levels both (i) at 0 months and (ii) 6 months post vaccination in the vaccine group (n = 23). We found that the average antibody titre at 6 months post-vaccination in PCR negative and PCR positive animals was significantly greater than at 0 months (Fig 1; PCR negative *p* = 0.002; PCR positive *p* < 0.001; S1 Table) indicating that we had successfully induced a vaccination-specific immune response.

### *Chlamydia* prevalence

Overall, the 60 animals initially included in our study had a *C*. *pecorum* prevalence of 54% at time of capture (as defined by *C*. *pecorum* species-specific PCR). After recruitment into the trial, the koalas were assigned into groups consisting of: (i) animals with a current infection, as defined by being *C*. *pecorum* PCR positive, at the Oc site (Vaccinated: n = 10; Control: n = 6); (ii) *C*. *pecorum* PCR positive animals at the UGT site (Vaccinated: n = 8; Control: n = 13); and (iii) animals that were *C*. *pecorum* PCR negative at either site (Vaccinated: n = 16; Control n = 21). Some animals (Vaccinated: n = 7; Control n = 6) were necessarily included in both groups due to the occurrence of *C*. *pecorum* positivity at both sites. At 12 months, the number of animals remaining in each group decreased (mortality among wild koalas) to: (i) *C*. *pecorum* PCR positive animals at the Oc site (Vaccinated: n = 7; Control: n = 4); (ii) *C*. *pecorum* PCR positive animals at the UGT site (Vaccinated: n = 5; Control: n = 6; and (iii) *C*. *pecorum* PCR negative animals (Vaccinated: n = 11; Control n = 10).

### Rate of new *Chlamydia* infections in vaccinated koalas compared to unvaccinated controls

For this analysis we utilized the animals that were *C*. *pecorum* negative at 0 months and able to be recaptured at 12 months. In the control group, the 12 month incidence rate at the UGT site was 25% (two new infections in the 8 animals in this group), and 20% (2/10) at the Oc site. By comparison, the vaccinated animals had a slightly lower 12 month incidence rate of 20% (2/10) and 12% (1/8) at the UGT and Oc sites respectively. While the incidence rate in the vaccinated group was lower, the group size was small and hence the difference was not statistically significant (raw data *X*^2^ = 0.392, *p* > 0.999; percentage differences *X*^2^ = 2.381, *p* = 0.176).

### Changes in *Chlamydia* load following vaccination

For animals that were infected (PCR positive) at the time of recruitment, we measured their *Chlamydia* load by quantitative-PCR (qPCR) at 0, 6, and 12 months to evaluate the effect the vaccine had on the level of chlamydial shedding (Table 1). For the purposes of analysis, we grouped the animals into three categories, based on their PCR load change, whether the load decreased, stayed stable, or increased (ΔqPCR ≤ -100, -99–99, and ≥ 100 copies/μL respectively).

**Table 1:** Change in *Chlamydia* PCR load following vaccination: Percentage (and raw number calculations) of koalas that were *C*. *pecorum* positive at 0 months (i.e. at initial vaccination time), and then exhibited changes in their *C*. *percorum* load between either 0 and 6 months, or between 0 and 12 months, post vaccination. Statistically significant effects are shown in bold. Trending (*P* < 0.1) results indicated with *. Grey shading represents groups with more than expected (based on Pearson residuals) for significant results. The changes are categorized as decreasing, stable or increasing (ΔqPCR ≤ -100, -99–99, and ≥ 100 copies/μL respectively).

	Eye (0 vs. 6 months)			Eye (0 vs. 12 months)			UGT (0 vs. 6 months)			UGT (0 vs. 12 months)		
	Decrease	Stable	Increase	Decrease	Stable	Increase	Decrease	Stable	Increase	Decrease	Stable	Increase
Control	33% (2)	0% (0)	67% (4)	100% (4)	0% (0)	0% (0)	69% (9)	0% (0)	31% (4)	83% (5)	0% (0)	17% (1)
Vacc	50% (5)	40% (4)	10% (1)	71% (5)	29% (2)	0% (0)	88% (7)	12% (1)	0% (0)	100% (5)	0% (0)	0% (0)
*X*^*2*^	85.677			31.619 ^a^			45.299			16.458 ^a^		
*P*	< 0.001 (0.052 *)			**< 0.001 (0.496)**			< 0.001 (0.099*)			**< 0.001 (0.999)**		

^a^ analysis based on 2 x 2 contingency table Chi-square owing to no individuals with decreasing loads for both control and vaccinated koala

At 6 months post-vaccination, animals in the vaccine group were significantly more likely to decrease or stabilize their chlamydial load, whereas animals in the control group were significantly more likely to increase their load (Table 1). This effect was observed as a near significant trend (*p* > 0.01) using the conservative (raw) data and a significant effect based on the more sensitive (%) data. For example, at the ocular site 90% (9/10) of vaccinated animals decreased or stabilized their load, compared to the control group where only 33% (2/6) had decreasing or stabilizing loads (Table 1). Similarly, at the UGT site, 100% (8/8) of animals in the vaccinated group had decreasing or stabilizing loads compared to 69% (9/13) in the control group (Table 1).

At 12 months, the positive vaccine effect was maintained at the UGT site with 100% (5/5) of vaccinated animals showing a decrease in chlamydial load compared to 83% (5/6) in the control group (Table 1). We are cautious about drawing conclusions on the statistical significance of this owing to the difference of only a single individual. However importantly, throughout the entire study, not one animal in the vaccine group showed an increase at the UGT site. At the Oc site at 12 months, 100% (7/7) of vaccinated animals also decreased or stabilized their chlamydial load, although a similar trend (100% [4/4] decrease) was seen in the control group (Table 1). Again, we are highly cautious about interpreting the statistical significance of this based on the sample size. Overall, smaller sample sizes of koalas, owing to field mortalities, cause us to be cautious about statistical interpretation of results at 12 months.

### Progression to chlamydial disease

To investigate the impact that vaccination had on the progression of chlamydial disease, we compared the presence and absence of disease in vaccinated and control animals. Over the 12 months of the study, only 1 of 23 (4% of koalas) vaccinated animals developed clinical signs of chlamydial disease, whereas 4 of 27 (14.8%) control animals developed clinical disease over the same time period. Based on percentage differences, the control and vaccinated groups were significantly dissimilar (*X*^2^ = 7.037, *p* = 0.013), but the same result could not be observed in the raw data (*X*^2^ = 1.512, *p* = 0.363) owing to the sample size. The one vaccinated animal developed mild, sub-acute, chronic cystitis, was treated in care with the standard chloramphenicol dosage and released as healthy. Three of the four animals that developed disease in the control group developed cystitis and were treated; the final animal developed severe and extensive reproductive disease as well as severe chronic cystitis, and was euthanized.

## Discussion

We have, for the first time, examined the effect of a rMOMP based anti-chlamydial vaccine on chlamydial infection risk and outcome in free-ranging koalas. The vaccine induced a significant immune response in wild-caught koalas. The incidence of new *C*. *pecorum* infections was lower at both anatomical sites in vaccinated animals, despite not being statistically significant. Importantly, we also found that vaccinated koalas were more likely to have stable or decreasing *C*. *pecorum* PCR loads, and were also less likely to increase their chlamydial burdens at 6 months post-vaccination at both anatomical sites. At 12 months, this positive effect could still be observed in the vaccinated cohort, with no animals increasing their chlamydial loads at either anatomical site. However, we caution the low number of koalas at this time point made statistical inference unreliable. Lastly, we showed a positive effect for protecting against progression to disease in vaccinated animals.

Therapeutic vaccines are a promising new approach to enhance immunogenicity, and reduce viral and bacterial load in infected humans and animals [15]. Due to the difficulties associated with antibiotic treatment in the koala, a therapeutic vaccine may provide an important alternative to reduce infection. While antibiotics are curative in many cases of chlamydial disease, the therapeutic course is relatively long and labour intensive, often precluding its efficacy for the treatment of koala outside of the clinic. Therefore, a therapeutic vaccine provides a more practical solution for disease management at a non-captive population level, particularly if a single-dose vaccine were to be developed. In our current study, the positive therapeutic effect seen at both anatomical sites in the koala is a promising result for the development of a therapeutic chlamydial vaccine for this species. The loss of meaningful statistical inference at 12 months due to severe field mortalities is disappointing, masking our ability to confidently detect an effect at this time interval. This effect seems largely skewed by the four animals in the control group reducing their chlamydial ocular burden. When followed longitudinally up to two years, two of these four animals developed clinical signs of disease, whereas none of the vaccinated animals in that cohort developed any clinical signs (unpublished data).

While we did not observe a significant improvement in the risk of new infections in the vaccinated koalas, it was interesting to note nevertheless that asymptomatically infected control animals were more likely to advance to disease than asymptomatically infected vaccinated animals. Promisingly, over 12 months, only one vaccinated animal developed new disease symptoms (cystitis), whereas 4 animals in the non-vaccinated cohort succumbed to disease (cystitis n = 3 and reproductive disease n = 1). While beyond the scope of this manuscript, it is also promising to observe that available longitudinal data for the remaining animals at 18–24 months suggests that two (of 7; 28.6%) additional control animals contracted cystitis, whereas none of the vaccinated animals has yet succumbed to disease (0/6; 0%).

To conclude, the first field trial to date of this prototype koala chlamydial vaccine suggests that vaccinated *Chlamydia*-infected koalas have an improved infection outcome—an outcome that highlights the potential for the development of a therapeutic vaccination schedule for this species. This is especially promising given the small sample sizes, and the natural variability of an outbred population. In the koala, the main goal for population management from an ecological standpoint is maintaining health and young animal recruitment. Therefore, if a vaccine is able to lower or prevent increases of infection load, as well as to decrease the progression to disease, than this will have positive effects on population health and fecundity and may be an important tool in the management and conservation of the koala.

## Supporting Information

S1 Table Plasma IgG antibody titers of both (i) at 0 months (pre-vaccination) and (ii) 6 months post vaccination in koalas (n = 23) vaccinated with a recombinant Major Outer Membrane Protein (MOMP) vaccine adjuvanted with an Immunostimulating complex adjuvant (ISC). (XLSX)
